# How Secure Are Two-Way Ping-Pong and LM05 QKD Protocols under a Man-in-the-Middle Attack?

**DOI:** 10.3390/e23020163

**Published:** 2021-01-29

**Authors:** Mladen Pavičić

**Affiliations:** 1 Center of Excellence for Advanced Materials and Sensors, Research Unit Photonics and Quantum Optics, Institute Ruder Bošković, 10000 Zagreb, Croatia; mpavicic@irb.hr; 2 Nanooptics, Department of Physics, Humboldt-Universität zu Berlin, 12489 Berlin, Germany

**Keywords:** quantum cryptography, quantum key distribution, two-way communication, 03.67.Dd, 03.67.Ac, 42.50.Ex

## Abstract

We consider a man-in-the-middle attack on two-way quantum key distribution ping-pong and LM05 protocols in which an eavesdropper copies all messages in the message mode, while being undetectable in the mode. Under the attack there is therefore no disturbance in the message mode and the mutual information between the sender and the receiver is always constant and equal to one and messages copied by the eavesdropper are always genuine. An attack can only be detected in the control mode but the level of detection at which the protocol should be aborted is not defined. We examine steps of the protocol to evaluate its security and find that the protocol should be redesigned. We also compare it with the security of a one-way asymmetric BB84-like protocol in which one basis serves as the message mode and the other as the control mode but which does have the level of detection at which the protocol should be aborted defined.

## 1. Introduction

Quantum cryptography, in particular quantum key distribution (QKD) protocols, offers us, in contrast to the classical one, provably unbreakable communication based on the quantum physical properties of the information carriers [1,2,3]. So far, implementations were mostly based on the one-way BB84 protocol [4] which is unconditionally secure provided the quantum bit error rate (QBER) is low enough. However, QBER in BB84-like protocols might be high and since we cannot discriminate eavesdropper’s (Eve’s) bit flips from bit flips caused by noise in the line, the request of having QBER low enough for processing the bits is often difficult to satisfy. e.g., 4-state BB84 with more than 0.11 [5] and 6-state BB84 [6] with more than 0.126 [5] disturbances (*D*) have to be aborted (*D* is defined as the amount of polarization-flips caused by Eve, the maximum being 0.5). *D* includes the inherent QBER as well as possible Eve in the line. If Eve were the only cause of *D*, the mutual information between the sender (Alice) and Eve ($I_{AE}$) wound surpass the one between Alice and the receiver (Bob) ($I_{AB}$): $I_{AE}>I_{AB}$ for D>0.11,0.126, respectively.

Protocols using two-way quantum communications have also been proposed. Since they are less efficient versions of BB84 protocols, they have no meaningful advantage. Here we show that the security of some two-way protocols is vulnerable under a man-in-the-middle (MITM) attack. In particular, entangled photon two-way protocols based on two [7] (also called a ping-pong (pp) protocol) and four ($\Psi^{\mp },\Phi^{\mp }$) [8] Bell states, on the one hand and a single photon deterministic Lucamarini–Mancini (LM05) protocol, on the other [9,10]. Several varieties, modifications, and generalisations of the latter protocol are given in [11,12,13,14]. Two varieties were implemented in [15,16]. The former pp protocol was implemented by Ostermeyer and Walenta in 2008 [17] while the protocol with four Bell states cannot be implemented with linear optics elements [18,19]. In the aforementioned references various security estimations have been obtained.

In [20] Lu, Fung, Ma, and Cai provide a security proof of an LM05 deterministic QKD for the kind of attack proposed in [9,10]. Nevertheless, they claim it to be a proof of the unconditional security of LM05. In [21] Han, Yin, Li, Chen, Wang, Guo, and Han provide a security proof for a modified pp protocol and prove its security against collective attacks in noisy and lossy channel.

All previous elaborations of the security of two-way protocols assume that Eve attacks each signal twice, once on the way from Bob to Alice, and later on its way back from Alice to Bob, and that in doing so she disturbs the signal in the message mode.

However, there is another attack in which an undetectable Eve encodes Bob’s signals by mimicking Alice’s encoding of a decoy signal sent to her which we elaborate on in this paper. We consider the two-way deterministic QKD protocols under a MITM attack where, Eve—undetectable in the message mode (MM)—can acquire all the messages, meaning that there is no disturbance in the MM ($D_{\mathrm{MM}}$) at all. In the control mode (CM) there is a disturbance ($D_{\mathrm{CM}}$), but there is no critical *D* at which Alice and Bob should abort the protocol. The only way to delete bits of the raw key snatched by Eve is via privacy amplification and for disturbances close to $D_{\mathrm{CM}}=0.5$, when Eve is in the line all time, it seems impossible to distinguish whether Eve has or has not obtained the whole key. In order to verify that conjecture, we prove that the security proof carried out in [20] does not cover a MITM attack and that therefore cannot be called “unconditional”.

We also compare two-way protocols under a MITM attack with a recent one-way asymmetric BB84-like protocol [22] in which the {|0〉,|1〉} basis serves as MM and the {|±〉} basis as CM, under a MITM attack. The latter protocol resolves the problem of absence of inherent critical disturbance by introducing a predetermined threshold disturbance after which Alice and Bob abort the protocol. This makes the protocol conditionally secure and we propose a similar solution for the two-way protocols.

In Section 2 we present the protocols and MITM attacks on them. In Section 3 we discuss the security of two-way protocols and analyze their proof of unconditional security; we also compare properties of two-way protocols with those of the standard BB84 and the aforementioned asymmetrical BB84-like one. In Section 4 we present some concluding points and a summary of the results achieved in the paper.

## 2. Protocols and Attacks on Them

Ping-pong (pp) protocol is based on two Bell states [7]. Bob prepares entangled photons in one of the Bell states, sends one of the photons to Alice while keeping the other one in a quantum memory (qm). Alice either returns the photon as is or acts on it so as to put both photons into another Bell states. Bob combines the photon he receives from Alice with the one he kept in qm at a beam splitter (BS) to decode Alice’s messages. The messages are said to be sent in message mode (MM). There is also a control mode (CM) in which Alice measures Bob’s photon and announces her outcomes over a public channel.

The Bell basis used in the pp protocol consists of two Bell states

(1) $$\begin{array}{c} |\Psi^{\mp }\rangle =\frac{1}{\sqrt{2}}{(|H\rangle }_{1}{|V\rangle }_{2}\mp {|V\rangle }_{1}{|H\rangle }_{2}), \end{array}$$

where ${|H\rangle }_{i}$ (${|V\rangle }_{i}$), i=1,2, represent horizontal (vertical) polarized photon states.

Photon pairs in the state $|\Psi^{-}\rangle$ are generated by a down-converted entangled photon source. To send $|\Psi^{-}\rangle$ state Alice just returns her photon to Bob. To send $|\Psi^{+}\rangle$ she puts a half-wave plate ($\mathrm{HWP}(0^{\circ })$) in the path of her photon. The HWP changes the sign of the vertical polarization.

At Bob’s BS the photons in state $|\Psi^{-}\rangle$ will split and those in state $|\Psi^{+}\rangle$ will bunch together.

**Figure 1:**
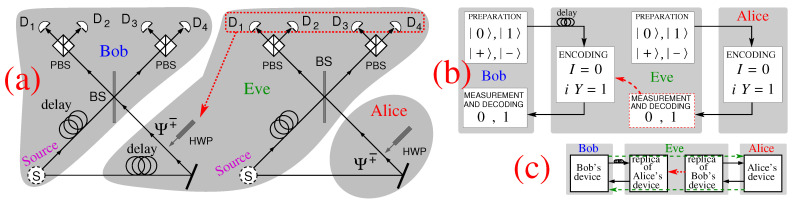
(**a**) Nguyen’s attack [23] by which Eve is able to deterministically (and undetectably in the message mode (MM)) copy every one of the Bell-state messages in the ping-pong (pp) protocol [7]; (**b**) Lucamarini’s attack ([24], p. 61, Figure 5.5) by which Eve is able to deterministically (and undetectably in the MM) copy every message in the LM05 protocol; (**c**) common schematics of both attacks; the green dashed line shows the path of photons when Eve is not in the line.

Eavesdropper Eve carries out an MITM, designed by Nguyen [23], as follows. She puts Bob’s photon in a qm (delays it in the fiber coil) and makes use of a copy of Bob’s device to send Alice a photon from a down-converted pair in state $|\Psi^{-}\rangle$ as shown in Figure 1a. When Eve receives the photon from Alice she combines it with the other photon from the pair and determines the Bell state in the same way Bob would. She uses this result to generate the same Bell state for Bob by putting the appropriate HWPs in the path of Bob’s photon.

Thus, Eve is able to copy every single message in the MM undetectably and therefore sending messages in the MM is equivalent to sending plain text “secured” by the CM.

In the LM05 protocol [9,24] Bob prepares a qubit in one of the four states |0〉, |1〉 (the Pauli Z eigenstates), |+〉, or |−〉 (Pauli X eigenstates) and sends it to his counterpart Alice. In the MM she modifies the qubit state by applying either I, which leaves the qubit unchanged and encodes the logical **0**, or by applying iY=ZX, which flips the qubit state and encodes the logical **1**. (iY|0〉=−|1〉, iY|1〉=|0〉, iY|+〉=|−〉, iY|−〉=−|+〉.) Alice now sends the qubit back to Bob who measures it in the same basis in which he prepared it and deterministically infers Alice’s operations, i.e., her messages, without basis reconciliation procedure.

Eavesdropper Eve carries out a MITM, designed by Lucamarini ([24], p. 61, Figure 5.5), as follows. As shown in Figure 1b. Eve delays Bob’s photon (qubit) in a fiber spool (a quantum memory) and sends her own decoy photon in one of the four states |0〉, |1〉, |+〉, or |−〉 to Alice, instead. Alice encodes her message via I or iY and sends the photon back. Eve measures it in the same basis in which she prepared it, reads off the message, encodes Bob’s delayed photon via I, if she read **0**, or via iY, if she read **1**, and sends it back to Bob.

Eve never learns the states in which Bob sent his photons but that is irrelevant in MM since only polarization flipping or not flipping encode messages. Alice also need not know Bob’s states [9]. Eve could only be revealed in CM in which Alice carries out a projective measurement of the qubit along a basis randomly chosen between Z and X, prepares a new qubit in the same state as the outcome of the measurement, sends it back to Bob, and reveals this over a classical public channel [9].

## 3. Security of the Protocols

To reach the main point of the paper, let us first discuss the one-way asymmetric (aBB84) and symmetric (sBB84, i.e., standard BB84) protocols.

A recent definition of aBB84 [25] reads: “Alice [asks her] entanglement-based source to [randomly] prepare quantum states in two bases, X={|0〉,|1〉} and $Z=\{(|0\rangle +|1\rangle )/\sqrt{2},(|0\rangle -\left|1\rangle \right)/\sqrt{2}\}$... Bob [randomly] measure[s] quantum systems in [these two] bases... The protocol is *asymmetric* [meaning that] the number of bits measured in the two bases (*n* bits in the X basis and *k* bits in the Z basis) are not necessarily equal [26]... **Sifting:** Alice and Bob broadcast their basis choices over the classical channel... **Error correction:** (EC) A reconciliation scheme that broadcasts [chosen] bits of classical error correction data is applied. Bob compute[s] an estimate $\hat{Y}$ of the raw key string Y. Alice computes universal${}_{2}$ hash function of Y [and] sends [it] to Bob. If the hash[es] of $\hat{Y}$ and Y disagree, the protocol aborts. **Privacy amplification:** (PA) Alice extracts *l* bits of secret key S from Y using a random universal${}_{2}$ hash function. The choice of function is communicated to Bob, who uses it to calculate S.” ([25], p. 3) There are other similar definitions of aBB84 in the literature [26,27,28,29,30].

When n=k, aBB84 turns into sBB84, i.e., it becomes identical to the original BB84. In what follows, when not explicitly stated otherwise, under BB84 we mean sBB84.

What is essential for the standard aBB84 and sBB84, is that Eve cannot avoid introducing disturbance (*D*). Specifically, when Alice sends messages in X and Z bases, Eve cannot avoid introducing *D*. E.g., Alice sends |1〉 in X basis, Eve reads it as $(|0\rangle +\left|1\rangle \right)/\sqrt{2}$ in Z basis and resends it to Bob who, say in X basis, reads it either as |0〉 or as |1〉. If the former, it will be discarded in the EC procedure, if the latter, it will be accepted as a valid message. As for Eve, no public information can enable her to find out what Bob actually measured hence she loses information.

**Figure 2:**
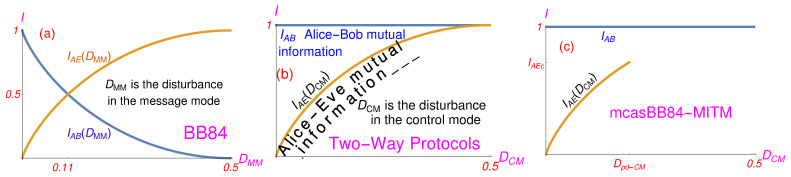
Mutual information plots for (**a**) one-way protocol BB84; (**b**) two-way protocols with either pp entangled Bell states or with LM05-like single photon states under a MITM attack; (**c**) one-way asymmetric BB84-like protocol, in which one basis serves as MM and the other as CM, under a MITM attack (mcasBB84-MITM); $I_{AEc}$ stands for $I_{AE}\left(D_{pd-\mathrm{CM}}\right)$.

However, Alice and Bob lose their information too, and as shown in Figure 2a in a BB84 protocol, when the level of disturbance approaches $D_{\mathrm{MM}}=0.11$ (the MM is the only mode of the standard BB84 protocol) the mutual information between Alice and Eve $I_{AE}$ approaches the mutual information between Alice and Bob $I_{AB}$ and they have to abort the protocol. Note that $I_{AB}=1+D\log_{2}D+\left(1-D\right)\log_{2}\left(1-D\right)$ and $I_{AE}=-D\log_{2}D-\left(1-D\right)\log_{2}\left(1-D\right)$ [31] and that, ideally, for $D_{\mathrm{MM}}<0.11$, EC can eliminate all errors induced by Eve and that PA can remove all key bits Eve might have collected, no matter how close to 0.11 $D_{\mathrm{MM}}$ is. This is so because both $I_{AB}$ and $I_{AE}$ are functions of $D_{\mathrm{MM}}$, i.e., functions of the disturbance in the message mode for which the mutual information in the very same message mode is calculated. The closer $D_{\mathrm{MM}}$ is to 0.11, the more difficult is for Alice and Bob, after PA, to extract the secure key from the raw key, since the former becomes smaller and smaller. “The efficiency of privacy amplification rapidly decreases when [$D_{\mathrm{MM}}$] increases” ([32], p. 165, mid right column). “At $D_{\mathrm{MM}}=0.11$ the secure-key length formally vanishes” ([33], p. 524). See also [31].

For a MITM attack on two-way protocols (which are without any sifting), when Eve is in the line all the time, there is no $D_{\mathrm{MM}}$ that Eve induces and the mutual information between Alice and Bob as well as between Alice and Eve is equal to unity: $I_{AB}=1$. In Figure 2b $D_{\mathrm{CM}}$ indicates the presence of Eve, where $D_{\mathrm{CM}}=0.5$ would mean that Eve is always in the line. For $D_{\mathrm{CM}}$ slightly below $D_{\mathrm{CM}}=0.5$ one cannot exclude the possibility that Eve has all the messages.

When Eve has all the messages then there is no Alice-Bob “privacy” they could amplify. When Eve snatches only a portion of messages, then, for $D_{\mathrm{CM}}$ close to 0.5, there is still a question whether Eve has all messages or not and whether Alice and Bob can erase Eve’s messages with their PA. With that in mind, we can examine the security evaluation for the MITM and verify whether the proofs of unconditional security carried out for other kind of attack on LM05 in [10,20] might apply to it as well.

In the aforementioned security proof [20], which is claimed to be unconditional, the authors assume that Eve probes the qubit, entangling it with an ancilla. However, their approach does not cover the MITM attacks. To show this we point to the following steps in their proof of unconditional security vs. two-way-protocol-under-MITM counter-steps:

[20] p. 2, 2nd paragraph from the top: “Alice announces partial of her key bits in the encoding mode [MM]. They compute the error rate *e* in the Alice–Bob channel.”
MITM *$I_{AB}=1$ and Eve does not induce any error in the* MM*, ever.*
[20] p. 2, Sec. III.A: “Eve cannot gain any information about Alice’s key bits if she only attacks the qubits after Alice’s encoding operation.”
MITM Since Eve in her MITM sends her own photons to Alice and then reads off I or iY from Alice’s encoding of those qubits, Eve gains all information from Alice’s qubits, more precisely, from Eve’s qubits encoded by Alice. Note the neither Alice nor Eve know which states the qubits Bob sends are in. They only control I and iY.
[20] Eve’s most general quantum operation can be described by a unitary operation together with an ancilla. In the Bob–Alice channel, when Bob sends a qubit in state |0〉 and Alice measures in the basis |0〉,|1〉, she will get the measurement outcomes |0〉 with probability $c_{00}^{2}$ or |1〉 with probability $c_{01}^{2}$.
MITM Alice does not measure qubits. She just applies I and iY.
[20] Eve’s most general attack (with ancillas) is
  $U_{BE}{|0\rangle }_{B}|E\rangle =c_{00}{|0\rangle }_{B}|E_{00}\rangle +c_{01}{|1\rangle }_{B}|E_{01}\rangle ,U_{BE}{|1\rangle }_{B}|E\rangle =c_{11}{|1\rangle }_{B}|E_{11}\rangle +c_{10}{|0\rangle }_{B}|E_{10}\rangle ,$
  $U_{BE}{|+\rangle }_{B}|E\rangle \;=\;c_{++}{|+\rangle }_{B}|E_{++}\rangle \;+\;c_{+-}{|-\rangle }_{B}|E_{+-}\rangle ,\;\;U_{BE}{|-\rangle }_{B}|E\rangle \;=\;c_{--}{|-\rangle }_{B}|E_{-+}\rangle \;+\;c_{-+}{|+\rangle }_{B}|E_{-+}\rangle .$
  Fidelities are $f_{0}=c_{00}^{2}$, $f_{1}=c_{11}^{2}$, $f_{+}=c_{++}^{2}$, and $f_{-}=c_{--}^{2}$. $f_{0}=f_{1}$ and $f_{-}=f_{+}$ are assumed...
  Bob’s qubit is in a mixed state $\rho^{B}=(|0\rangle \langle 0|+|1\rangle \langle 1|)/2$. The joint state of the forward qubit and Eve’s ancilla becomes $\rho_{BA}^{BE}=U_{BE}(\rho^{B}⊗|E\rangle \langle E|)U_{BE}$. Alice’s encoded qubit together with Eve’s ancillas is: $\rho^{ABE}=\frac{1}{2}{\left|0\rangle \langle 0\right|}^{A}⊗\rho_{BA}^{BE}+\frac{1}{2}{\left|1\rangle \langle 1\right|}^{A}⊗Y_{B}\rho_{BA}^{BE}Y_{B}$...
  The asymptotic key generation rate is $r=\lim_{m\to \infty }\frac{k(m)}{m}$, where *m* is the size of the raw key and k(m) is the number of the final key bits. Alice sends Bob EC information over a classical channel so that he can correct his raw key to match Alice’s.
MITM Eve does not induce any disturbance, so there is no EC.
[20] The final key is then derived by applying two-universal hashing to their common raw key as PA. The secure key rate $r_{\mathrm{PA}}$ for secret key generation is bounded by the conditional entropy of Alice and Bob’s key bits given the quantum information of Eve about the key bits $r_{\mathrm{PA}}=S\left(\rho^{A}|\rho^{BE}\right)=-tr\rho^{ABE}\log_{2}\rho^{ABE}+\mathrm{tr}\rho^{BE}\log_{1}\rho^{BE}=1-h\left(\xi \right)$, where $\xi =c_{++}^{2}-c_{1}^{2}$, $c_{1}=c_{01}=c_{10}$, and $h\left(x\right)=-x\log_{2}x-\left(1-x\right)\log_{2}\left(1-x\right)$ is the binary Shannon entropy. In particular, if Eve does not attack the forward qubits in the Bob-Alice channel, i.e., $f_{0}=f_{1}=f_{+}=f_{-}=1$, one can find that $r_{\mathrm{PA}}\left(\xi \right)=1$. This states that Eve cannot gain any information about Alice’s key bits if she does not attack the travel qubit in the Bob–Alice channel first. Consider the case that Eve measures each forward qubit in the Bob-Alice channel in the basis |0〉,|1〉. Alice and Bob can verify that $f_{0}=f_{1}=1$, and $f_{+}=f_{-}=\frac{1}{2}$. In this case, we have $r_{\mathrm{PA}}\left(\xi \right)=0$. On the other hand, Eve can also measure each forward qubit in the Bob-Alice channel in the basis |+〉,|−〉, which gives $f_{+}=f_{-}=1$ and $f_{0}=f_{1}=\frac{1}{2}$, and thus $r_{\mathrm{PA}}\left(\xi \right)=0$. That is, Eve can gain full information of Alice’s key bits if she has exactly known the forward states before Alice’s encoding operations.
MITM *Eve does not measure qubits (or ancillas). When she is in the line all the time, she just reads off I and iY Alice executed on her qubits and applies them to Bob’s qubits she stored, i.e., she copies the whole key—both sides have the whole key. There is no privacy which can be amplified. That means we have $r_{\mathrm{PA}}=1$, not 0. This deserves a clarification. $r_{\mathrm{PA}}=\lim_{m\to \infty }\frac{k(m)}{m}=1$ states that the secret key is equivalent to the raw key in the infinite limit for both Alice and Bob and Eve, what corresponds to $I_{AB}=I_{AE}\left(D_{Max-\mathrm{CM}}\right)=1$, for $D_{Max-\mathrm{CM}}=0.5$. So, $k_{\mathrm{PA}}\left(m\right)$ should not be used as a secret key, but that does not mean that we can infer $k_{\mathrm{PA}}\left(m\right)=0$. After* PA *both parties have the same $r_{\mathrm{PA}}=1$ and discarding $k_{\mathrm{PA}}\left(m\right)$ does not turn $r_{\mathrm{PA}}$ to zero. Discarding the key is based on Alice and Bob’s estimation from the* CM*, i.e., from outside of the* MM *space of calculation. The way of calculating $k_{\mathrm{PA}}\left(m\right)$ so as to include discarding of estimated bits both parties might possess should follow from an adequately elaborated* PA *procedure and its algorithms. A starting step should be a predefined $D_{Max-\mathrm{CM}}<0.5$ and its inclusion in the protocol via $I_{MaxAE}=I_{AE}\left(D_{Max-\mathrm{CM}}\right)$. That would give us a conditional security of the protocol.*

Taken together, the analysis carried out in [20] is applicable and correct for the attacks on two-way protocols in which Eve reads off the states of qubits with the help of ancillas but is inapplicable to MITM attacks. Therefore, their proof is not universal, i.e., cannot be considered unconditional.

Can Alice and Bob still achieve reliable security of their two-way protocol? To answer this let us first compare one-way (e.g., BB8) and two-way protocols.

Under standard attacks on one way protocols, Eve is left with less and less information about the key when she approaches the critical disturbance $D_{crit-\mathrm{MM}}=0.11$, i.e., the messages she snatches end up scrambled, up to 50% of the time. However, Eve also scrambles Bob and Alice’s messages so that after PA, half of the messages are deleted and half coincide. So, neither party is left with any usable bit.

In two-way protocols under MITM it is different. Eve does not scramble Bob and Alice’s messages at all at higher and higher values of $D_{\mathrm{CM}}$ and the longer she is in the line the more messages she copies; for $D_{Max-\mathrm{CM}}=0.5$ their secret keys are identical and no bare PA (hashing only) can change that.

However, with two-way protocols, when Eve is not in the line all the time, Alice and Bob carry out the PA, guided by the level of disturbance, i.e., the error rate in the CM. The standard PA uses a binary string of obtained messages to produce a new shorter strings via universal hashing. Alice randomly chooses a (permutation) function $f:{\left\{0,1\right\}}^{m}\to {\left\{0,1\right\}}^{k(m)}$ among some universal${}_{2}$ class of functions. She then sends both f(x) and a description of *f* to Bob via universal hashing. After computing f(y), where *y* is his corresponding string, Bob checks whether it agrees with f(x). If it does, a basic property of universal hashing allows them to assume that x=y ([34], p. 214).

The problem emerges with this version of the PA, i.e., with algorithms it makes use of, because Alice and Bob should be able to estimate the length k(m) of the secure key with respect to the length of the raw key *m* which would guarantee them that Eve is not in possession of a single bit of k(m), but via their bare (“blind”) PA they always get x=y, i.e., they do not have a benchmark for estimating the amount of bits Eve lost. The PA procedures elaborated in the literature do not help since they are made for one-way protocols (BB84, B92, etc.) and are rather involved and intricate—c.f. [35]. We have not found a PA procedure elaborated for two-way protocols in the literature. What makes it challenging is the asymptotic approach of $I_{AE}\left(D_{\mathrm{CM}}\right)$ to $I_{AB}=1$ shown in Figure 2b, which is absent in the BB84 protocol—see Figure 2a, on the one hand, and the high *D*, on the other. Whether we can find an efficient PA algorithm for two-way protocols remains to be seen.

A special kind of an aBB84-like protocol in which the X basis serves as MM and Z as CM proposed by Bunandar et al. [22] can help us to better understand the problem of unlimited *D*. We call the protocol a message-control-(a)symmetric BB84 (mcasBB84) protocol. In Table 1 we compare the properties of the BB84, two-way, and mcasBB84 protocols under MITM attacks.

Let us consider a MITM attack on mcasBB84 (mcasBB84-MITM) which Eve carries out so as to measure and resend all qubits in the X basis. Since Eve receives only |0〉 and |1〉 messages and resends them unchanged, she does not introduce any disturbance in the MM and $I_{AB}=1$. Eve’s $I_{AE}$ rises with her increased presence in the line. If she were in the line all the time, we would have $D_{\mathrm{CM}}=0.5$ and $I_{AE}=1$. However, the protocol does not allow that. Instead, it predetermines a threshold value $D_{pd\mathrm{CM}}$ and if $D_{\mathrm{CM}}>D_{pd\mathrm{CM}}$ Alice and Bob will abort it as specified by Bunandar et al. ([22], p. 7). The protocol is an adaptation of the three-state aBB84 protocol which makes use of both X and Z bases for MM as in the standard BB84 only with two additional decoy settings put forward by Lim, Curty, Walenta, Xu and Zbinden [36] which itself builds on other decoy-state pioneering methods as, e.g., the one proposed by Wang [37]. The idea of a predetermined threshold value $D_{pd\mathrm{CM}}$ is taken over from [30]. $D_{pd\mathrm{CM}}$ serves [22] to calculate a conditional security. The calculation determines which maximal $D_{pd\mathrm{CM}}$ is acceptable for an implementation. Apart from solving two-way-protocol maximal *D* problem, the mcasBB84-MITM has another big advantage (with respect to exponential attenuation of photons in optical fibres) that its photons cover the same distance as the original BB84 (*L*), i.e., half the distance LM05 photons cover (2*L*) and a quarter of the distance pp photons cover (4*L*).

## 4. Conclusions

To summarize, we considered man-in-middle (MITM) attacks on two kinds of two-way QKD protocols (pp with entangled photons and LM05 with single photons) in which an undetectable Eve can decode all the messages in the message mode (MM) and showed that the mutual information between Alice and Bob is not a function of disturbance in the MM, since there is no disturbance in the MM, but is equal to unity no matter whether Eve is in the line or not. Eve induces a disturbance ($D_{\mathrm{CM}}$) only in the control mode (CM). In a way, Alice’s sending of the key is equivalent to sending an unencrypted plain text (via photons obtained by and returned to Bob) secured by an indicator of Eve’s presence. That burdens the protocols with the following drawbacks under a MITM attack:

- the photons must cover the double distance than in an equivalent one-way BB84-like protocol (mcasBB84) which also has analogous MM and CM modes;
- while the BB84 protocol is unconditionally secure, and its revised version, the macasBB84 protocol proposed recently is only conditionally secure, the proof of unconditional security of the LM05 protocol given in [20] is not valid, as shown in details in Section 3; the mcasBB84 protocol has a predetermined threshold value of the CM disturbance ($D_{pdCM}$) at which Bob and Alice must abort the protocol whilst the considered two-way protocols do not have such a critical CM disturbance at which to abort the protocol;
- since there are no errors in the MM mode, the privacy amplification (PA) is the only way to establish the security of the protocols and again in contrast to the mcasBB84 no PA procedures for the two-way protocols have been provided in the literature;

Let us elaborate on these point in the reverse order.

In the two-way protocols the mutual information between Alice and Bob is always greater than or equal to the one between Alice and Eve. When they are equal, i.e., when Eve is in the line all the time, then Alice and Bob and Eve have identical messages and there is no privacy which can be amplified and PA cannot erase key bits Eve has snatched. For a $D_{\mathrm{CM}}<0.5$, but close to 0.5, Alice and Bob do not have a procedure and algorithms to obtain the secret key of which Eve possess almost all bits. Note that $I_{AE}\left(D_{\mathrm{CM}}\right)$ approaches $I_{AE}=1$ asymptotically and that a maximal $D_{CM}$ after which Alice and Bob have to abort the protocol is not defined.

This is related to our analysis (in Section 3) of the security proof given in [20] which the authors call unconditional. In the analysis in Section 3 we show that their proof does not cover the man-in-the-middle attack (MITM) and that therefore cannot be called “unconditional.”

To better understand the problem of absence of a maximal tolerable $D_{\mathrm{CM}}$ (after which Alice and Bob have to abort the protocol) in two-way protocols, in Section 3 we compare protocol with a newly proposed one-way asymmetric BB84-like protocol [22] (mcasBB84) in which X basis serves as MM and Z basis as CM under a MITM attack (mcasBB84-MITM). We show that mcasBB84-MITM without defined maximal $D_{\mathrm{CM}}$ would be completely equivalent to two-way protocols under MITM. However, the mcasBB84 protocol resolves the problem of a maximal $D_{\mathrm{CM}}$ by means of a predetermined threshold value $D_{pd\mathrm{CM}}$. When $D_{\mathrm{CM}}>D_{pd\mathrm{CM}}$ Alice and Bob abort the protocol ([22], p. 7). The security calculated for such $D_{pd\mathrm{CM}}$ ([22], pp. 7–10), i.e., an elaborated PA procedure, can be called a “conditional security.” An additional advantage (with respect to exponential attenuation of photons in optical fibres) of mcasBB84 is that photons do not travel from Bob to Alice and back to Bob, but only from Alice to Bob (see Table 1).

A similar solution for two-way protocols would be to redesign the protocol so as to either calculate a critical $D_{crit-\mathrm{CM}}$ at which Alice and Bob would be able to erase all bits Eve might have possessed via privacy amplification (PA) or to predetermine threshold value of the disturbance in the CM, $D_{pd-\mathrm{CM}}$, for which PA calculations might be carried out. The former calculation, if possible, would provide us with an unconditionally security and the latter one would provide us with a conditional security of the protocols. How to do either of the calculations is an open question, but we conjecture that the former calculation is not feasible.

## Figures and Tables

**Table 1:** Properties of a symmetric BB84-like protocol under an arbitrary attack compared with properties of pp, LM05, and  asymmetric mcasBB84 protocols under MITM. For the pp and LM05 protocols D<0.5 means that Eve is in the line only a portion of the time and D=0.5 that she is in the line all the time. $D_{pd-\mathrm{CM}}$ is a predetermined threshold value of D<0.5 for the mcasBB84-MITM [22] protocol.

	BB84	pp	LM05	mcasBB84-MITM
mode(s)	MM	MM + CM	MM + CM	MM + CM
disturbance	$0\leq D_{\mathrm{MM}}\leq 0.5$	$D_{\mathrm{MM}}=0$ $0\leq D_{\mathrm{CM}}\leq 0.5$	$D_{\mathrm{MM}}=0$ $0\leq D_{\mathrm{CM}}\leq 0.5$	$D_{\mathrm{MM}}=0$ $0\leq D_{\mathrm{CM}}\leq D_{pd-\mathrm{CM}}$
maximal disturbance	$D_{critical-\mathrm{MM}}=0.11$	?	?	$D_{pd-\mathrm{CM}}$
secure	for $D_{\mathrm{MM}}<0.11$	for $D_{\mathrm{CM}}<\;?$	for $D_{\mathrm{CM}}<\;?$	for $D_{\mathrm{CM}}<D_{pd-\mathrm{CM}}$
mutual information	$I_{AB}\left(D_{\mathrm{MM}}\right)$, $I_{AE}\left(D_{\mathrm{MM}}\right)$	$I_{AB}=1$ $0\leq I_{AE}\left(D_{\mathrm{CM}}\right)<1$	$I_{AB}=1$ $0\leq I_{AE}\left(D_{\mathrm{CM}}\right)<1$	$I_{AB}=1$ $0\leq I_{AE}\left(D_{\mathrm{CM}}\right)<I_{AE}\left(D_{pd-\mathrm{CM}}\right)$
photon distance	*L*	4*L*	2*L*	*L*
trans-mittance	T	$T^{4}$	$T^{2}$	T
